# The heavy burden of female reproduction. A commentary on: ‘Time for a change: patterns of sex expression, health and mortality in a sex-changing tree’

**DOI:** 10.1093/aob/mcz117

**Published:** 2019-08-10

**Authors:** John R Pannell

**Affiliations:** Department of Ecology and Evolution, University of Lausanne, Lausanne, Switzerland

## Abstract

This article comments on:

**Jennifer Blake-Mahmud and Lena Struwe. 2019**. Time for a change: patterns of sex expression, health and mortality in a sex-changing tree. *Annals of Botany***124(3)**: 367–377.

Gender and sex expression in plants varies from purely male, through hermaphroditic or monoecious (where plants have bisexual or unisexual flowers, respectively), to purely female. These sex or gender categories provide useful labels, but plant sexuality is very often a quantitative trait, and the labels are often inadequate descriptors of the complex mating strategies that plants actually express (Lloyd, 1980). In many dioecious populations, females and males are ‘inconstant’ or ‘leaky’ in their sex expression, frequently producing sex organs or flowers of the opposite sexual function (Lloyd and Bawa, 1984). Hermaphrodites or monoecious individuals, too, vary in the extent to which they emphasize one or other of their two sexual functions. In gynodioecious populations, for instance, in which females co-occur with hermaphrodites, the hermaphrodites by necessity must act more as fathers than mothers, and should have male-biased sex allocation. Nevertheless, the very same hermaphrodites would act equally as male and female in a population lacking females, illustrating the fact that the functional gender of an individual depends as much on that of its potential mates as on its own sex allocation (Lloyd, 1980). In a paper published in this issue of *Annals of Botany*, Blake-Mahmud and Struwe (2019) highlight another feature of plant reproduction that is missed by categorical labels of gender: that long-lived plants may also vary their sex expression from one reproductive season to the next, to the point of switching sex completely.

Blake-Mahmud and Struwe (2019) studied the sex expression of individuals of a population of the long-lived maple *Acer pensylvanicum*, recording their sex over a period of four successive years (Fig. 1A and B). In any one year, the population might be labelled ‘trioecious’, comprising males, females and hermaphrodites. However, the authors report that, over the course of their study, a full 54% of the trees they followed switched sex between years, several more than once. Apart from recording a switch in sex between the categories male, female and monoecious, the study also recorded a large number of monoecious individuals that changed their relative allocation to the two sexual functions, underscoring the important point that gender is a quantitative trait in *A. pensylvanicum*, as in many flowering plants, and not a strictly categorical one. *Acer pensylvanicum* does not have a trimodal gender distribution, and is thus not really trioecious.

**Fig. 1.**
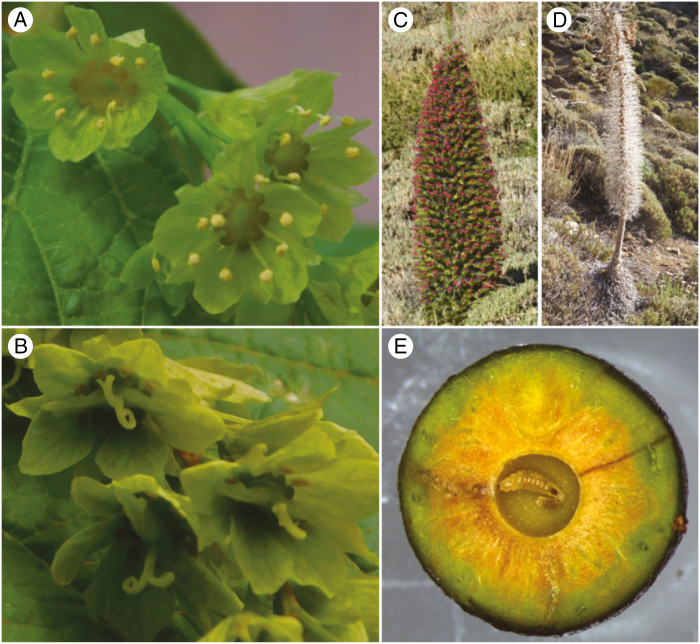
Images of (A) part of a male inflorescence and (B) part of a female inflorescence of *Acer pensylvanicum*. Images (C) and (D) illustrate an inflorescence and a dead plant after flowering of the monocarpic perennial Canary Islands endemic *Echium wildpretii*. Flowering in *E. wildpretii* drains the plant of resources and causes its death. Image (E) illustrates gall-wasp larvae in a cut-open gall of *Acacia longifolia*. The wasp induces the plant to invest resources to protect it from predators, compromising the plant’s growth and survival. Images (A) and (B) courtesy of Jennifer Blake-Mahmud. Images (C) and (D) taken by the author. Image (E) courtesy of Marie Henriksen.

A particularly interesting aspect of Blake-Mahmud and Struwe’s (2019) study was their assessment of various features of plant growth, size and health that were associated with shifts in sex expression, including transitions between flowering and not flowering, and between living and dead. While their results are complex in their details, a number of clear patterns emerge. Most striking was the tendency (1) for trees that died during the course of the study to have flowered as largely or purely female in the preceding season, (2) for plants in a female state to be more likely to show signs of poor health, such as crown damage, and (3) for stem elongation to be substantially lower for plants flowering as female than as male. In discussing their results, the authors acknowledge that the correlational patterns they report do not allow cause and effect to be disentangled, but their results are nevertheless consistent with the idea that a greater burden of reproduction falls on plants that produce not only flowers but also fruits.

The suggestion that a plant’s female function exacts a greater cost of reproduction than its male function is not new (e.g., Obeso, 2002) and would seem to stand to reason: fruiting is expensive. Similar conclusions have been reached in many other studies reporting differences in life history between the sexes, but there have also been illuminating exceptions. For example, in the dioecious wind-pollinated herb *Mercurialis annua*, males prevented from flowering enjoyed a much greater boost to their growth than did females treated similarly (Harris and Pannell, 2008), pointing to a steeper trade-off between reproduction and growth in males than females, and males of *M. annua* senesce sooner, in contrast to *A. pensylvanicum*. Similarly, in the South African fynbos shrub *Leucadendron xanthoconus*, males that invested particularly heavily in flowering experienced greater mortality (Bond and Maze, 1999). In general, biased sex ratios in long-lived dioecious species provide indirect evidence for different costs of reproduction, with one sex suffering greater mortality than the other (Field *et al.*, 2013). Although female-biased sex ratios are not uncommon, male-biased ratios are more frequent, hinting at a greater cost of reproduction for females, as implied by Blake-Mahmud and Struwe’s (2019) results, too. What stands out in their study is their ability to model the various factors that are associated with allocating to male versus female functions.

It is easy to be satisfied with the explanation that reproduction through one sex can be costlier to a plant than through the other, but a moment’s reflection will show that it need not be so. A decision to flower as a female, for instance, implies a commitment towards paying potentially heavy costs of fruiting later in the season, but one could easily imagine females simply producing fewer flowers to ease that burden, or maturing fewer fruits per flower. Indeed, both males and females could in principle adjust their reproductive effort to minimize its impact on growth, tissue maintenance or future reproduction. The fact that females often do adopt a strategy that compromises their survival reminds us that natural selection does not act to minimize mortality, but to maximize fitness. Monocarpic perennial species, which flower only once and then die, provide a particularly striking example of a selected strategy with ultimately lethal consequences (Fig. 1C and D). However, the more subtle association between female flowering and mortality reported by Blake-Mahmud and Struwe (2019) illustrates the same point, as do sex-ratio biases in dioecious species mentioned above.

Why might females not modulate their reproductive investment to reduce risks of mortality more than they do? Two ideas come to mind. First, the pattern hints at the possibility of so-called accelerating fitness gain curves, whereby increases in reproductive effort reap increasingly handsome rewards in terms of the production of successful progeny (West, 2009). For example, larger investment in fruit production may attract increasingly more frugivorous seed dispersers, scattering progeny over a greater area and thus reducing how much they need to compete with one another to become established. The shape of fitness gain curves has been invoked as crucial for explaining the evolutionary stability of combined versus separate sexes and other strategies (West, 2009), yet gain curves have been very difficult to estimate because they require knowledge of the fate of dispersed pollen or seeds. Studies such as that of Blake-Mahmud and Struwe (2019) provide an indirect signal that they might indeed sometimes be accelerating. If so, it would help to explain why so many individuals of *A. pensylvanicum* frequently adopt a strategy of unisexuality, which theory shows should be favoured by accelerating gain curves.

Second, could it be that the reproductive investment of females is decided not by the females themselves, but by heavy claims made on females by the seeds and fruits they produce, i.e., that females are coerced into allocating more to reproduction than is optimal for them? The idea of an allocation conflict between parents and their progeny is well established (Wilkins and Haig, 2003), and it is tempting to think that the results presented by Blake-Mahmud and Struwe (2019) point in that direction too. Should we consider seeds and fruits developing on a tree in somewhat the same way as the growth of galls induced by wasps that induce the plant to invest vast amounts of resource towards the protection of the wasps’ eggs and developing grubs (Fig. 1E)? The potentially fatal consequences of such investment are the basis for the managed introduction of gall wasps into populations of several invasive *Acacia* species around the world as biological controls. When attempting to understand reproductive investment in plant populations, we might do well to pose old question: *cui bono*?
